# Homicides of American Indians/Alaska Natives — National Violent Death Reporting System, United States, 2003–2018

**DOI:** 10.15585/mmwr.ss7008a1

**Published:** 2021-11-19

**Authors:** Emiko Petrosky, Laura M. Mercer Kollar, Megan C. Kearns, Sharon G. Smith, Carter J. Betz, Katherine A. Fowler, Delight E. Satter

**Affiliations:** ^1^Division of Violence Prevention, National Center for Injury Prevention and Control, CDC; ^2^Office of Tribal Affairs and Strategic Alliances, Center for State, Tribal, Local and Territorial Support, CDC

## Abstract

**Problem/Condition:**

Homicide is a leading cause of death for American Indians/Alaska Natives (AI/ANs). Intimate partner violence (IPV) contributes to many homicides, particularly among AI/AN females. This report summarizes data from CDC’s National Violent Death Reporting System (NVDRS) on AI/AN homicides. Results include victim and suspect sex, age group, and race/ethnicity; method of injury; type of location where the homicide occurred; precipitating circumstances (i.e., events that contributed to the homicide); and other selected characteristics.

**Period Covered:**

2003–2018.

**Description of System:**

NVDRS collects data regarding violent deaths obtained from death certificates, coroner/medical examiner reports, and law enforcement reports and links related deaths (e.g., multiple homicides and homicide followed by suicide) into a single incident. This report includes data on AI/AN homicides that were collected from 34 states (Alabama, Alaska, Arizona, California, Colorado, Georgia, Illinois, Indiana, Iowa, Kansas, Kentucky, Louisiana, Maine, Maryland, Massachusetts, Michigan, Minnesota, Missouri, Nebraska, Nevada, New Jersey, New Mexico, New York, North Carolina, Ohio, Oklahoma, Oregon, Pennsylvania, Rhode Island, South Carolina, Utah, Virginia, Washington, and Wisconsin) and the District of Columbia.

**Results:**

NVDRS collected data on 2,226 homicides of AI/ANs in 34 states and the District of Columbia during 2003–2018. The age-adjusted AI/AN homicide rate was 8.0 per 100,000 population. The homicide rate was three times higher in AI/AN males than females (12.0 versus 3.9), and the median age of AI/AN victims was 32 years (interquartile range: 23–44 years). Approximately half of AI/AN homicide victims lived or were killed in metropolitan areas (48.2% and 52.7%, respectively). A firearm was used in nearly half (48.4%) of homicides and in a higher percentage of homicides of AI/AN males than females (51.5% versus 39.1%). More AI/AN females than males were killed in a house or apartment (61.8% versus 53.7%) or in their own home (47.7% versus 29.0%). Suspects were identified in 82.8% of AI/AN homicides. Most suspects were male (80.1%), and nearly one third (32.1%) of suspects were AI/ANs. For AI/AN male victims, the suspect was most often an acquaintance or friend (26.3%), a person known to the victim but the exact nature of the relationship was unclear (12.3%), or a relative (excluding intimate partners) (10.5%). For AI/AN female victims, the suspect was most often a current or former intimate partner (38.4%), an acquaintance or friend (11.5%), or a person known to the victim but the exact nature of the relationship was unclear (7.9%). A crime precipitated 24.6% of AI/AN homicides (i.e., the homicide occurred as the result of another serious crime). More AI/AN males were victims of homicides due to an argument or conflict than females (54.7% versus 37.3%), whereas more AI/AN females were victims of homicides due to IPV than males (45.0% versus 12.1%). For homicides related to IPV, 87.2% of AI/AN female victims were killed by a current or former intimate partner, whereas approximately half (51.5%) of AI/AN male victims were corollary victims (i.e., victims killed during an IPV-related incident who were not the intimate partners themselves).

**Interpretation:**

This report provides a detailed summary of NVDRS data on AI/AN homicides during 2003–2018. Interpersonal conflict was a predominant circumstance, with nearly half of all AI/AN homicides precipitated by an argument and for female victims, 45.0% precipitated by IPV.

**Public Health Action:**

NVDRS provides critical and ongoing data on AI/AN homicides that can be used to identify effective and early intervention strategies for preventing these deaths. When possible, violence prevention efforts should include community-developed, culturally relevant, and evidence-based strategies. These efforts should incorporate traditional native knowledge and solutions, implement and possibly adapt evidence-based IPV and other violence prevention strategies, and consider the influence of historical and larger societal factors that increase the likelihood of violence in AI/AN communities.

## Introduction

Missing and Murdered Indigenous People (MMIP)* is an issue that has gained federal attention (*1*–*3*). In 2019, homicide was the fifth leading cause of death for American Indian/Alaska Native (AI/AN) males and the seventh leading cause of death for AI/AN females aged 1–54 years (*4*). The Presidential Task Force on Missing and Murdered American Indians and Alaska Natives, also known as Operation Lady Justice, was established in 2019 to enhance the operation of the criminal justice system and to address the concerns of AI/AN communities regarding MMIP (*1*). In 2020, Savanna’s Act (*2*) was passed to increase U.S. governmental agency coordination to reduce violent crimes within tribal lands and against AI/ANs, and the Not Invisible Act (*3*) directed the U.S. Department of Justice to review, revise, and develop law enforcement and justice protocols to address MMIP. According to the U.S. Department of Justice, the rate of violent crime against AI/ANs (101 violent crimes per 1,000 AI/ANs) was more than twice the rate of the general U.S. resident population (41 per 1,000 persons) during 1992–2002 (*5*). AI/ANs also experience higher rates of adverse childhood experiences, including child abuse and neglect and family and community violence, than other racial/ethnic groups (*6*–*8*), which increase their risk for other forms of violence, such as homicide. The risk factors for violence among AI/ANs are compounded by multiple and multilayered traumas, including historical (e.g., war and loss of land, language, access to traditional ways, and cultural identity), intergenerational (e.g., child and elder abuse and neglect), and ongoing (e.g., racism and structural inequities) traumas (*9*–*12*).

AI/ANs also have reported high levels of intimate partner violence (IPV) (*13*), defined as physical or sexual violence, stalking, or psychological harm by a current or former intimate partner or spouse (*14*). According to National Intimate Partner and Sexual Violence Survey data collected during 2010–2012, 47.5% of non-Hispanic AI/AN women and 40.5% of non-Hispanic AI/AN men have experienced contact sexual violence, physical violence, or stalking by an intimate partner during their lifetime (*13*). Similar to women of other racial/ethnic groups, approximately half of AI/AN female homicides are related to IPV (*15*). National Violent Death Reporting System (NVDRS) data during 2003–2014 indicate that IPV contributed to 55.4% of homicides among non-Hispanic AI/AN women (*15*).

In 2019, an estimated 6.9 million persons, representing approximately 2% of the U.S. population, reported AI/AN ancestry either alone or in combination with one or more other races (*16*). Approximately 75% of AI/ANs live in urban, suburban, and rural settings outside of reservations (*17*). The 10 states with the largest AI/AN populations in 2019 were Arizona, California, Oklahoma, New Mexico, Texas, North Carolina, Alaska, Washington, South Dakota, and New York (*17*). Currently, there are 574 federally recognized Indian tribes (*18*) and approximately 100 state-recognized tribes in the United States (*17*), each representing distinct communities with diverse histories and cultures, and 324 federally recognized American Indian reservations, including federal reservations and off-reservation trust land (*16*). Tribes are sovereign nations with distinct political communities and territorial boundaries, within which their authority is exclusive, and are subordinate to the federal government but not to state governments (*19*).

The first step in preventing violence against AI/ANs is defining the problem (*20*). Accurate and comprehensive surveillance data help identify risk factors for violence and can be used to guide culturally relevant public health interventions. Further, when developing prevention programs, it is important to consider the circumstances of AI/AN homicides and how they might differ across tribes and from other populations. Tribal communities have requested studies on AI/AN homicide to guide prevention efforts (*21*), and a key activity for the Presidential Task Force on Missing and Murdered American Indians and Alaska Natives is to review AI/AN homicide data (*1*). However, previous studies have been limited by racial misclassification, which can underestimate violence and homicide prevalence in AI/AN populations (*22*), and the diverse population sizes of AI/AN persons are often too small to provide estimates, which can lead to the “statistical invisibility” of these groups (*23*). These limitations make it difficult to define fatal interpersonal violence among AI/ANs and, in turn, might restrict attention and resources to prevent these deaths.

This report presents data from NVDRS to better understand the circumstances surrounding AI/AN homicides and to highlight the contextual factors that place AI/ANs at risk for homicide. Because of the high percentage of homicides related to IPV among AI/AN women, this report further examines data on IPV-related AI/AN homicides. This report also recommends community approaches to protect AI/AN persons from homicide and IPV using a violence prevention framework that incorporates complex and contextual knowledge of AI/AN communities (*20*). The findings in this report can be used by tribes; federal, state, and local governments; public health and health services sectors; the criminal justice system; and victim services to guide violence prevention efforts.

## Methods

### National Violent Death Reporting System

NVDRS is an active, state-based surveillance system that collects information from death certificates, coroner/medical examiner reports, and law enforcement reports on the characteristics and circumstances of violent deaths, including homicides (*24*). NVDRS combines information for each death and links deaths that are related (e.g., multiple homicides and homicide followed by suicide) into a single incident. Trained state-level data abstractors code up to 600 variables in NVDRS using standardized guidance from CDC and enter the variables into an NVDRS web-based system. These variables include characteristics of victims and suspected perpetrators (suspects), incidents (e.g., when and where the incident occurred), weapons that inflicted fatal injuries, and circumstances that directly contributed to the death (e.g., IPV). Data on these precipitating circumstances often originate from investigators’ interviews with informants who knew the victim, witnessed the incident, or both.

State participation in NVDRS has expanded over time (*25*). NVDRS data have been collected in Maryland, Massachusetts, New Jersey, Oregon, South Carolina, and Virginia since 2003; Alaska, Colorado, Georgia, North Carolina, Oklahoma, Rhode Island, and Wisconsin since 2004; Kentucky, New Mexico, and Utah since 2005; Ohio since 2011; Michigan since 2014; Arizona, Connecticut, Hawaii, Illinois, Indiana, Iowa, Kansas, Maine, Minnesota, New Hampshire, New York, Pennsylvania, Vermont, and Washington since 2015; and Alabama, California, Delaware, District of Columbia, Louisiana, Missouri, Nebraska, Nevada, and West Virginia since 2017. Three states reported data on a subset of counties that represented at least 80% of violent deaths in their state during 2016–2018 (Illinois and Pennsylvania) and 2016–2017 (Washington; statewide since 2018). California reported 2017 data from four counties (Los Angeles, Sacramento, Shasta, and Siskiyou) and 2018 data from 21 counties (Amador, Butte, Fresno, Humboldt, Imperial, Kern, Kings, Lake, Los Angeles, Marin, Mono, Placer, Sacramento, San Benito, San Diego, San Francisco, San Mateo, Shasta, Siskiyou, Ventura, and Yolo). Data from Illinois, Pennsylvania, Washington, and California are not representative of all violent deaths that occurred in these four states because <100% of violent deaths were reported. Hawaii only provided data during 2015–2016 because of lack of complete data (<50% of cases had circumstance information from the coroner, medical examiner, or law enforcement report) in other years.

### Definitions

NVDRS defines homicide as a death resulting from the use of physical force or power, threatened or actual, against another person, group, or community when a preponderance of evidence indicates that the use of force was intentional (*26*). Homicide was classified by the *International Classification of Diseases, 10th Revision* cause-of-death codes X85–X99, Y00–Y09, Y87.1, and U01–U02 (*27*). Victims and suspects were classified as AI/AN if they had ancestries of the original inhabitants of North America who maintained their cultural identification through tribal affiliation or community recognition^†^ (*26*). Precipitating circumstances are defined as the events that contributed to the infliction of a fatal injury and are reported on the basis of the content of coroner/medical examiner and law enforcement investigative reports (Appendix) (*26*). Data abstractors select from a list of potential circumstances and are required to code all circumstances that are known to relate to each incident; therefore, circumstances are not mutually exclusive. If either the coroner/medical examiner report or law enforcement report indicates the presence of a circumstance, then the abstractor endorses the circumstance (e.g., if the law enforcement report indicated that a physical fight between two persons resulted in the death of a victim, then the circumstance variable “physical fight” is endorsed). Certain circumstances are coded to a specific manner of death (e.g., “drug involvement” is collected for homicides); other circumstances are coded across all manners of death (e.g., “argument or conflict” led to the victim’s death). If circumstances are unknown (e.g., a body was found in the woods with no other details reported), the data abstractor does not endorse circumstances, and these deaths are then excluded from the denominator for circumstance values.

IPV-related deaths were defined as those involving intimate partner homicide (i.e., victim was an intimate partner or spouse [current, former, or unspecified] of the suspect); corollary victims of IPV-related homicide (i.e., other deaths associated with IPV, including homicides of victims who were not the intimate partner, such as family, friends, others who intervened in IPV, first responders, and bystanders); or homicides precipitated by jealousy or distress over an intimate partner’s relationship or suspected relationship with another person. Detailed descriptions of all variables collected by NVDRS are available in the NVDRS Coding Manual (*26*).

### Analysis 

This report summarizes AI/AN homicide data from 34 states and the District of Columbia that participated in NVDRS during 2003–2018 (all available years). Six states (Connecticut, Delaware, Hawaii, New Hampshire, Vermont, and West Virginia) had no AI/AN homicides during 2003–2018 and were not included in this analysis. Therefore, the AI/AN homicide data in this report are from 30 states that collected statewide data, four states that collected data from a subset of counties (Illinois, Pennsylvania, Washington, and California), and the District of Columbia. Analyses were conducted for all AI/AN homicides and also the subset of IPV-related AI/AN homicides. Homicide rates were calculated using intercensal and postcensal bridged-race population estimates compiled by CDC’s National Center for Health Statistics and were age-adjusted to the 2010 standard U.S. population (*28*). Rural-Urban Commuting Area codes were used to classify geographic areas into metropolitan and nonmetropolitan categories.^§^ From all data captured by NVDRS, variables relevant to homicides were selected for analysis. Descriptive analyses of sociodemographic characteristics of victims and suspects (i.e., age, race/ethnicity, education, metropolitan status, and pregnancy status), mechanisms used to inflict fatal injuries, and incident characteristics (i.e., location of injury and victim’s relationship to the suspect) were conducted. Categories of precipitating circumstances included victim’s mental health and substance use, interpersonal problems and conflict (e.g., IPV and family relationship problem), life stressors (e.g., crisis during previous or upcoming 2 weeks), crime and criminal activity (e.g., drug involvement), and other events of the homicide that were relevant to the death (e.g., victim used a weapon). All descriptive analyses were conducted using SAS (SAS Institute).

## Results

### Characteristics of Homicide Victims

NVDRS collected data on 2,226 AI/AN homicides (1,681 male victims and 545 female victims) in 34 states (30 states collecting statewide data and a subset of California, Illinois, Pennsylvania, and Washington counties) and the District of Columbia during 2003–2018. A total of 5.7% of AI/AN homicide incidents involved multiple victims (8.4% of homicides of female and 4.8 of males). The age-adjusted AI/AN homicide rate was 8.0 per 100,000 population and was three times higher among males than females (12.0 versus 3.9). The median age of AI/AN victims was 32 years (interquartile range: 23–44 years). More than one fourth (27.5%) of AI/AN victims were aged 25–34 years; 10.3% were children aged ≤17 years (Table 1). Three fourths (74.8%) of AI/AN victims were of a single race; 6.7% were Hispanic or Latino. For victims who were of multiple races, 15.6% were AI/AN and non-Hispanic White, 4.2% were AI/AN and non-Hispanic Black, and 1.3% were AI/AN and non-Hispanic Asian or Native Hawaiian or other Pacific Islander (NHOPI). Among AI/AN victims aged ≥18 years, 14.7% had attended some college or more. Pregnancy status was known for 88 (25.7%) of 343 female victims of reproductive age (15–44 years); among these, 14.8% were pregnant or within 6 weeks postpartum at the time of death.

**TABLE 1:** Number and percentage of American Indian/Alaska Native homicides, by victim’s sex and selected demographic and incident characteristics — National Violent Death Reporting System,* 2003–2018

Characteristic	Male (n = 1,681) No. (%)^†^	Female (n = 545) No. (%)	Total (n = 2,226) No. (%)
**Age group (yrs)**			
<1	36 (2.1)	22 (4.0)	**58 (2.6)**
1–9	51 (3.0)	49 (9.0)	**100 (4.5)**
10–17	52 (3.1)	20 (3.7)	**72 (3.2)**
18–24	327 (19.5)	95 (17.4)	**422 (19.0)**
25–34	486 (28.9)	127 (23.3)	**613 (27.5)**
35–44	317 (18.9)	109 (20.0)	**426 (19.1)**
45–54	240 (14.3)	57 (10.5)	**297 (13.3)**
55–64	103 (6.1)	41 (7.5)	**144 (6.5)**
≥65	69 (4.1)	25 (4.6)	**94 (4.2)**
**Ethnicity**			
Hispanic or Latino	112 (6.7)	37 (6.8)	**149 (6.7)**
**Education^§^**			
< High school graduate or GED certificate equivalent	470 (30.5)	118 (26.0)	**588 (29.5)**
High school graduate or GED certificate equivalent	553 (35.9)	148 (32.6)	**701 (35.1)**
Some college or more	200 (13.0)	93 (20.5)	**293 (14.7)**
Unknown	319 (20.7)	95 (20.9)	**414 (20.7)**
**Pregnancy status^¶^**			
Pregnant or ≤6 weeks postpartum	—	13 (14.8)	**—**
Metropolitan status**			
Metropolitan resident	788 (48.4)	256 (47.8)	**1,044 (48.2)**
Metropolitan injury location	755 (53.3)	232 (50.8)	**987 (52.7)**
**Method of injury**			
Firearm	865 (51.5)	213 (39.1)	**1,078 (48.4)**
Sharp instrument	368 (21.9)	99 (18.2)	**467 (21.0)**
Blunt instrument	142 (8.4)	66 (12.1)	**208 (9.3)**
Personal weapons (e.g., hands, feet, or fists)	145 (8.6)	61 (11.2)	**206 (9.3)**
Hanging/strangulation/suffocation	37 (2.2)	35 (6.4)	**72 (3.2)**
Other method††	67 (4.0)	44 (8.1)	**111 (5.0)**
Unknown	57 (3.4)	27 (5.0)	**84 (3.8)**
**Location of injury**			
House/apartment	902 (53.7)	337 (61.8)	**1,239 (55.7)**
Street/highway	273 (16.2)	39 (7.2)	**312 (14.0)**
Natural area	90 (5.4)	49 (9.0)	**139 (6.2)**
Motor vehicle	71 (4.2)	25 (4.6)	**96 (4.3)**
Parking lot/public garage/public transport	53 (3.2)	9 (1.7)	**62 (2.8)**
Other location^§§^	209 (12.4)	53 (9.7)	**262 (11.8)**
Unknown	83 (4.9)	33 (6.1)	**116 (5.2)**
**Injured at victim’s home**			
Victim’s home	488 (29.0)	260 (47.7)	**748 (33.6)**

**Abbreviation:** GED = general education development.

* NVDRS data have been collected in Maryland, Massachusetts, New Jersey, Oregon, South Carolina, and Virginia since 2003; Alaska, Colorado, Georgia, North Carolina, Oklahoma, Rhode Island, and Wisconsin since 2004; Kentucky, New Mexico, and Utah since 2005; Ohio since 2011; Michigan since 2014; Arizona, Connecticut, Hawaii, Illinois, Indiana, Iowa, Kansas, Maine, Minnesota, New Hampshire, New York, Pennsylvania, Vermont, and Washington since 2015; and Alabama, California, Delaware, District of Columbia, Louisiana, Missouri, Nebraska, Nevada, and West Virginia since 2017. Three states reported data on a subset of counties that represented at least 80% of violent deaths in their state during 2016–2018 (Illinois and Pennsylvania) and 2016–2017 (Washington). California reported 2017 data from four counties (Los Angeles, Sacramento, Shasta, and Siskiyou) and 2018 data from 21 counties (Amador, Butte, Fresno, Humboldt, Imperial, Kern, Kings, Lake, Los Angeles, Marin, Mono, Placer, Sacramento, San Benito, San Diego, San Francisco, San Mateo, Shasta, Siskiyou, Ventura, Yolo). Hawaii provided data only for 2015–2016 because of lack of complete data in other years. ^†^ Percentages might not total 100% due to rounding. ^§^ Percentage is based on the number of homicide decedents aged ≥18 (n = 1,996; 1,542 males and 454 females). ^¶^ Percentage is based on the number of female decedents of reproductive age (15–44 years) with known pregnancy status (n = 88). ** Percentages are based on the number of homicide decedents with a known residence (n = 2,164 [97.2%]; 1,628 males [96.8%] and 536 females [98.3%]) and injury location (n = 1,874 [84.2%]; 1,417 males [84.3%] and 457 females [83.9%]). Zip Code Rural-Urban Commuting Area (RUCA) codes (2010) were used to determine whether decedents resided in nonmetropolitan versus metropolitan areas. RUCA codes measure daily commuting flows, population density, and urbanization levels to classify subcounty level geographic areas. Victim residential Zip codes were dichotomized as “metro” (RUCA codes 1–3) and “nonmetro” (RUCA codes 4–10). Descriptions of the RUCA classification codes 1–10 are available at https://www.ers.usda.gov/data-products/rural-urban-commuting-area-codes/documentation. ^††^ Other method includes (in descending order): motor vehicles (e.g., buses, motorcycles, other transport vehicles), fire/burns, drowning, fall, poisoning, intentional neglect, and other (single method). ^§§^ Other location includes (in descending order): commercial/retail area, hotel/motel, park/playground/sports or athletic area, bar/nightclub, jail/prison, abandoned house/building/warehouse, synagogue/church/temple, hospital or medical facility, office building, industrial or construction area, farm, preschool/school/college/school bus, bridge, supervised residential facility, railroad tracks, and other unspecified location.

Approximately half of AI/AN homicide victims lived or were killed in metropolitan areas (48.2% and 52.7%, respectively) (Table 1). A firearm was used in nearly half (48.4%) of homicides and in a higher percentage of homicides among AI/AN males than females (51.5% versus 39.1%). A sharp instrument was used in 21.0% of homicides. More AI/AN females than males were killed in a house or apartment (61.8% versus 53.7%) or a natural area (e.g., field or river) (9.0% versus 5.4%), whereas more AI/AN males than females were killed on a street or highway (16.2% versus 7.2%). More AI/AN females than males were killed in their own home (47.7% versus 29.0%).

### Characteristics of Homicide Suspects

A suspect was identified in 82.8% of AI/AN homicides (81.1% of homicides of males and 87.9% of females) (Table 2). The age, sex, and race/ethnicity of the suspect were known in 69.6%, 90.8%, and 71.7% of cases, respectively. Nearly one fourth (21.6%) of suspects were aged 25–34 years and 20.1% were aged 18–24 years. Most suspects were male (80.1%). Nearly one third (32.1%) were AI/AN, 39.6% were non-AI/AN, and the race/ethnicity of the remaining 28.3% were unknown. Half (51.8%) of non-AI/AN suspects were non-Hispanic White, 28.4% were non-Hispanic Black, 16.3% were Hispanic (any race except AI/AN), and 3.6% were non-Hispanic Asian or NHOPI. The victim’s relationship to the suspect was known in 76.4% of homicides (73.8% of male victims and 83.9% of female victims). For AI/AN male victims, the suspect was most often an acquaintance or friend (26.3%), a person known to the victim but the exact nature of the relationship was unclear (12.3%), or a relative (excluding intimate partners [10.5%]). For AI/AN female victims, the suspect was most often a current or former intimate partner (38.4%), an acquaintance or friend (11.5%), or a person known to the victim but the exact nature of the relationship was unclear (7.9%).

**TABLE 2:** Number and percentage of American Indian/Alaska Native homicides, by victim’s sex, selected demographics of homicide suspects, and the victim’s relationship to the suspect — National Violent Death Reporting System,* 2003–2018

Characteristic	Male (n = 1,364) No. (%)^†^	Female (n = 479) No. (%)	Total (n = 1,843) No. (%)
**Suspect age group (yrs)**			
<18	72 (5.3)	10 (2.1)	**82 (4.4)**
18–24	284 (20.8)	86 (18.0)	**370 (20.1)**
25–34	276 (20.2)	122 (25.5)	**398 (21.6)**
35–44	151 (11.1)	83 (17.3)	**234 (12.7)**
45–54	93 (6.8)	47 (9.8)	**140 (7.6)**
55–64	33 (2.4)	12 (2.5)	**45 (2.4)**
≥65	9 (<1.0)	5 (1.0)	**14 (<1.0)**
Unknown	446 (32.7)	114 (23.8)	**560 (30.4)**
**Suspect sex**			
Male	1,090 (79.9)	386 (80.6)	**1,476 (80.1)**
Female	135 (9.9)	62 (12.9)	**197 (10.7)**
Unknown	139 (10.2)	31 (6.5)	**170 (9.2)**
**Suspect race/ethnicity**			
AI/AN	429 (31.5)	162 (33.8)	**591 (32.1)**
Not AI/AN	527 (38.6)	203 (42.4)	**730 (39.6)**
White, non-Hispanic	264 (50.1)	114 (56.2)	**378 (51.8)**
Black, non-Hispanic	160 (30.4)	47 (23.2)	**207 (28.4)**
Hispanic or Latino, any race except AI/AN	90 (17.1)	29 (14.3)	**119 (16.3)**
Asian/Pacific Islander, non-Hispanic	13 (2.5)	13 (6.4)	**26 (3.6)**
Unknown, non-Hispanic	408 (29.9)	114 (23.8)	**522 (28.3)**
**Victim's relationship to suspect^§^**			
Acquaintance/friend	359 (26.3)	55 (11.5)	**414 (22.5)**
Spouse/intimate partner (current or former)	71 (5.2)	184 (38.4)	**255 (13.8)**
Other person, known to victim	168 (12.3)	38 (7.9)	**206 (11.2)**
Other relative	143 (10.5)	34 (7.1)	**177 (9.6)**
Stranger	122 (8.9)	22 (4.6)	**144 (7.8)**
Child	60 (4.4)	30 (6.3)	**90 (4.9)**
Other relationship^¶^	83 (6.1)	39 (8.1)	**122 (6.6)**
Unknown	358 (26.2)	77 (16.1)	**435 (23.6)**

**Abbreviation:** AI/AN = American Indian Alaska Native.

* NVDRS data have been collected in Maryland, Massachusetts, New Jersey, Oregon, South Carolina, and Virginia since 2003; Alaska, Colorado, Georgia, North Carolina, Oklahoma, Rhode Island, and Wisconsin since 2004; Kentucky, New Mexico, and Utah since 2005; Ohio since 2011; Michigan since 2014; Arizona, Connecticut, Hawaii, Illinois, Indiana, Iowa, Kansas, Maine, Minnesota, New Hampshire, New York, Pennsylvania, Vermont, and Washington since 2015; and Alabama, California, Delaware, District of Columbia, Louisiana, Missouri, Nebraska, Nevada, and West Virginia since 2017. Three states reported data on a subset of counties that represented at least 80% of violent deaths in their state during 2016–2018 (Illinois and Pennsylvania) and 2016–2017 (Washington). California reported 2017 data from four counties (Los Angeles, Sacramento, Shasta, and Siskiyou) and 2018 data from 21 counties (Amador, Butte, Fresno, Humboldt, Imperial, Kern, Kings, Lake, Los Angeles, Marin, Mono, Placer, Sacramento, San Benito, San Diego, San Francisco, San Mateo, Shasta, Siskiyou, Ventura, Yolo). Hawaii provided data for 2015–2016 because of lack of complete data in other years. ^†^ Percentages might not total 100% due to rounding and are based on the number of homicide decedents with a known suspect (n = 1,843 [82.8%]; 1,364 males [81.1%] and 479 females [87.9%]). ^§^ The following sentence can be used as a general guide for interpreting victim-suspect relationship: “The victim is the ____________ of the suspect.” For example, when a parent kills a child, the relationship is “Child” not “Parent” (“The victim is the child of the suspect”). The sentence is a general guide and some relationships might not be captured by this sentence (e.g., other person known to victim or victim was law enforcement officer killed in the line of duty). ^¶^ Other relationship includes (in descending order): parent, child of suspect’s boyfriend/girlfriend (e.g., child killed by mother’s boyfriend), rival gang member, intimate partner of suspect's parent (e.g., teenager kills his mother’s boyfriend), and victim was a law enforcement officer injured in the line of duty.

### Precipitating Circumstances of Homicides

Precipitating circumstances were identified in 83.2% of homicides (Table 3). An argument or conflict precipitated half (50.3%) of AI/AN homicides and preceded a larger percentage of homicides of AI/AN males than females (54.7% versus 37.3%). A crime precipitated 24.6% of homicides (i.e., the homicide occurred as the result of another serious crime), and the crime was in progress at the time of the fatal injury in 66.6% of these incidents. The type of crime most frequently precipitating homicides of AI/AN males was assault or homicide (35.3%), robbery (32.4%), burglary (16.5%), and drug trade (15.3%) and for AI/AN females was assault or homicide (35.8%), rape or sexual assault (25.7%), robbery (19.3%), burglary (9.2%), and drug trade (9.2%). More homicides of AI/AN females than males were related to IPV (45.0% versus 12.1%). Other common precipitating circumstances included a physical fight between two persons (23.3%), which precipitated more homicides of AI/AN males than females (27.7% versus 10.9%), and drug involvement (10.9%). Homicide victims were reported to have had a problem with substance use, alcohol, or both in 27.8% of cases; 17.8% of victims had a noted problem with substances other than alcohol and 14.1% with alcohol. Although less common, 4.3% of homicide victims were reported to have been currently diagnosed with a mental health problem and 1.2% had a current depressed mood at the time of death; 1.8% of victims were reported to be currently receiving mental health treatment.

**TABLE 3:** Number and percentage of American Indian/Alaska Native homicides, by victim’s sex and circumstance of the homicide — National Violent Death Reporting System,* 2003–2018

Circumstance^†^	Male No. (%)^§^	Female No. (%)	Total No. (%)
**Total homicides**	**1,681**	**545**	**2,226**
**Homicides with known circumstances (% of total)**	**1,384 (82.3)**	**469 (86.1)**	**1,853 (83.2)**
**Mental health/Substance use** ^¶^			
Substance use problem (excluding alcohol)	183 (17.6)	64 (18.4)	247 (17.8)
Alcohol problem	159 (15.3)	36 (10.4)	195 (14.1)
Current diagnosed mental health problem	43 (4.1)	17 (4.9)	60 (4.3)
History of ever being treated for a mental health problem	35 (3.4)	14 (4.0)	49 (3.5)
Current mental health treatment	19 (1.8)	6 (1.7)	25 (1.8)
Current depressed mood	10 (<1.0)	6 (1.7)	16 (1.2)
Other addiction (e.g., gambling or sex)	3 (<1.0)	1 (<1.0)	4 (<1.0)
**Interpersonal**			
Intimate partner violence–related	167 (12.1)	211 (45.0)	378 (20.4)
Family relationship problem^¶^	97 (9.3)	26 (7.5)	123 (8.9)
Other relationship problem (nonintimate)	123 (8.9)	22 (4.7)	145 (7.8)
Jealousy	55 (4.0)	30 (6.4)	85 (4.6)
Victim of interpersonal violence during past month	18 (1.7)	32 (9.2)	50 (3.6)
Perpetrator of interpersonal violence during past month	32 (3.1)	4 (1.2)	36 (2.6)
**Life stressor**			
Argument or conflict	757 (54.7)	175 (37.3)	932 (50.3)
Physical fight (two persons, not a brawl)**	204 (27.7)	28 (10.9)	232 (23.3)
Crisis during previous or upcoming 2 weeks	85 (6.1)	36 (7.7)	121 (6.5)
History of child abuse/neglect	10 (1.4)	6 (2.3)	16 (1.6)
**Crime and criminal activity**			
Precipitated by another crime	346 (25.0)	109 (23.2)	455 (24.6)
Crime in progress^††^	228 (65.9)	75 (68.8)	303 (66.6)
Drug involvement	157 (11.3)	45 (9.6)	202 (10.9)
Gang related	89 (6.4)	12 (2.6)	101 (5.5)
Terrorist attack	0 (0.0)	0 (0.0)	0 (0.0)
**Homicide event**			
Victim used a weapon	115 (8.3)	5 (1.1)	120 (6.5)
Caretaker abuse/neglect led to death**	28 (3.8)	34 (13.2)	62 (6.2)
Brawl	63 (4.6)	7 (1.5)	70 (3.8)
Random violence^¶^	35 (3.4)	7 (2.0)	42 (3.0)
Justifiable self-defense	55 (4.0)	0 (0.0)	55 (3.0)
Drive-by shooting^¶^	33 (3.2)	8 (2.3)	41 (3.0)
Mentally ill suspect^§§^	26 (1.9)	19 (4.0)	45 (2.4)
Walk-by assault**	19 (2.6)	3 (1.2)	22 (2.2)
Victim was a bystander	23 (1.7)	13 (2.8)	36 (1.9)
Victim was an intervener assisting a crime victim	15 (1.1)	7 (1.5)	22 (1.2)
Victim was a police officer on duty	5 (<1.0)	0 (0.0)	5 (<1.0)
Stalking**	0 (0.0)	1 (<1.0)	1 (<1.0)
Prostitution**	0 (0.0)	1 (<1.0)	1 (<1.0)
Hate crime	1 (<1.0)	0 (0.0)	1 (<1.0)
Mercy killing	0 (0.0)	0 (0.0)	0 (0.0)

* NVDRS data have been collected in Maryland, Massachusetts, New Jersey, Oregon, South Carolina, and Virginia since 2003; Alaska, Colorado, Georgia, North Carolina, Oklahoma, Rhode Island, and Wisconsin since 2004; Kentucky, New Mexico, and Utah since 2005; Ohio since 2011; Michigan since 2014; Arizona, Connecticut, Hawaii, Illinois, Indiana, Iowa, Kansas, Maine, Minnesota, New Hampshire, New York, Pennsylvania, Vermont, and Washington since 2015; and Alabama, California, Delaware, District of Columbia, Louisiana, Missouri, Nebraska, Nevada, and West Virginia since 2017. Three states reported data on a subset of counties that represented at least 80% of violent deaths in their state during 2016–2018 (Illinois and Pennsylvania) and 2016–2017 (Washington). California reported 2017 data from four counties (Los Angeles, Sacramento, Shasta, and Siskiyou) and 2018 data from 21 counties (Amador, Butte, Fresno, Humboldt, Imperial, Kern, Kings, Lake, Los Angeles, Marin, Mono, Placer, Sacramento, San Benito, San Diego, San Francisco, San Mateo, Shasta, Siskiyou, Ventura, Yolo). Hawaii provided data only for 2015–2016 because of lack of complete data in other years. ^†^ Includes homicides with one or more circumstances. Total numbers do not equal the sums of the columns because more than one circumstance could have been present per decedent. ^§^ Percentage is based on the number of homicide decedents with known circumstances. ^¶^ Variable collected for homicides since 2009. Denominator is homicides with known circumstances during 2009–2018 (n = 1,385; 1,038 males and 347 females). ** Variable collected for homicides since 2013. Denominator is homicides with known circumstances during 2013–2018 (n = 995; 737 males and 258 females). ^††^ Denominator includes those decedents involved in an incident that was precipitated by another crime. ^§§^ Percentage is based on the number of homicide decedents with a known suspect (n = 1,843 [82.8%]; 1,364 males [81.1%] and 479 females [87.9%]).

### Characteristics of IPV-Related Homicide Victims

NVDRS collected data on 380 AI/AN homicides precipitated by IPV (167 male victims and 213 female victims) in 34 states (30 states collecting statewide data and a subset of Illinois, Pennsylvania, Washington, and California counties) and the District of Columbia during 2003–2018 (Table 4). A total of 7.4% of IPV-related homicides involved multiple victims in the incident (10.3% of homicides of females and 3.6% of males). Approximately one fourth of AI/AN victims of IPV-related homicide were aged 25–34 years (26.1%) or 35–44 years (25.8%) and 6.1% were children aged <17 years. Few (5.3%) AI/AN IPV-related homicide victims were Hispanic or Latino. Approximately one fifth (20.2%) of AI/AN victims of IPV-related homicide aged ≥18 years attended some college or more. Pregnancy status was known for 42 (27.6%) of 152 female victims of reproductive age (15–44 years); among these, 14.3% were pregnant or within 6 weeks postpartum at the time of death.

**TABLE 4:** Number and percentage of American Indian/Alaska Native intimate partner violence-related homicides,* by victim’s sex and selected demographic and incident characteristics — National Violent Death Reporting System,† 2003–2018

Characteristic	Male (n = 167) No. (%)^§^	Female (n = 213) No. (%)	Total (n = 380) No. (%)
Age group (yrs)			
<1	0 (0.0)	1 (<1.0)	**1 (<1.0)**
1–9	6 (3.6)	9 (4.2)	**15 (3.9)**
10–17	3 (1.8)	4 (1.9)	**7 (1.8)**
18–24	27 (16.2)	37 (17.4)	**64 (16.8)**
25–34	50 (29.9)	49 (23.0)	**99 (26.1)**
35–44	35 (21.0)	63 (29.6)	**98 (25.8)**
45–54	28 (16.8)	32 (15.0)	**60 (15.8)**
55–64	10 (6.0)	14 (6.6)	**24 (6.3)**
≥65	8 (4.8)	4 (1.9)	**12 (3.2)**
**Ethnicity**			
Hispanic or Latino	9 (5.4)	11 (5.2)	**20 (5.3)**
**Education^¶^**			
< High school graduate or GED certificate equivalent	37 (23.4)	43 (21.6)	**80 (22.4)**
High school graduate or GED certificate equivalent	60 (38.0)	59 (29.6)	**119 (33.3)**
Some college or more	23 (14.6)	49 (24.6)	**72 (20.2)**
Unknown	38 (24.1)	48 (24.1)	**86 (24.1)**
Pregnancy status**			
Pregnant or ≤6 weeks postpartum	—	6 (14.3)	—
**Metropolitan status^††^**			
Metropolitan resident	71 (43.6)	95 (45.0)	**166 (44.4)**
Metropolitan injury location	63 (44.1)	90 (47.6)	**153 (46.1)**
**Method of injury**			
Firearm	77 (46.1)	85 (39.9)	**162 (42.6)**
Sharp instrument	56 (33.5)	36 (16.9)	**92 (24.2)**
Personal weapons (e.g., hands, feet, or fists)	12 (7.2)	29 (13.6)	**41 (10.8)**
Blunt instrument	10 (6.0)	25 (11.7)	**35 (9.2)**
Hanging/strangulation/suffocation	4 (2.4)	21 (9.9)	**25 (6.6)**
Other method^§§^	7 (4.2)	11 (5.2)	**18 (4.7)**
Unknown	1 (<1.0)	6 (2.8)	**7 (1.8)**
**Location of injury**			
House/apartment	129 (77.2)	147 (69.0)	**276 (72.6)**
Street/highway	20 (12.0)	14 (6.6)	**34 (8.9)**
Natural area	4 (2.4)	13 (6.1)	**17 (4.5)**
Other location^¶¶^	13 (7.8)	35 (16.4)	**48 (12.6)**
Unknown	1 (<1.0)	4 (1.9)	**5 (1.3)**
**Injured at victim’s home**			
Victim’s home	79 (47.3)	124 (58.2)	**203 (53.4)**

**Abbreviation:** GED = general education development.

* Includes victims killed by an intimate partner (e.g., current, former, or unspecified spouse, boyfriend, or girlfriend), victims killed during an intimate partner violence-related homicide who were not the intimate partner of the suspect (e.g., family, friends, and others who might have intervened in intimate partner violence, such as first responders or bystanders), and homicides precipitated by jealousy or distress over an intimate partner’s relationship or suspected relationship with another person. ^†^ NVDRS data have been collected in Maryland, Massachusetts, New Jersey, Oregon, South Carolina, and Virginia since 2003; Alaska, Colorado, Georgia, North Carolina, Oklahoma, Rhode Island, and Wisconsin since 2004; Kentucky, New Mexico, and Utah since 2005; Ohio since 2011; Michigan since 2014; Arizona, Connecticut, Hawaii, Illinois, Indiana, Iowa, Kansas, Maine, Minnesota, New Hampshire, New York, Pennsylvania, Vermont, and Washington since 2015; and Alabama, California, Delaware, District of Columbia, Louisiana, Missouri, Nebraska, Nevada, and West Virginia since 2017. Three states reported data on a subset of counties that represented at least 80% of violent deaths in their state during 2016–2018 (Illinois and Pennsylvania) and 2016–2017 (Washington). California reported 2017 data from four counties (Los Angeles, Sacramento, Shasta, and Siskiyou) and 2018 data from 21 counties (Amador, Butte, Fresno, Humboldt, Imperial, Kern, Kings, Lake, Los Angeles, Marin, Mono, Placer, Sacramento, San Benito, San Diego, San Francisco, San Mateo, Shasta, Siskiyou, Ventura, Yolo). Hawaii provided data only for 2015–2016 because of lack of complete data in other years. ^§^ Percentages might not total 100% due to rounding. ^¶^ Percentage is based on the number of intimate partner violence-related homicide decedents aged ≥18 years (n = 357; 158 males and 199 females). ** Percentage is based on the number of intimate partner violence-related female decedents of reproductive age (15–44 years) with known pregnancy status (n = 42). ^††^ Percentages are based on the number of homicide decedents with a known residence (n = 374 [98.4%]; 163 males [97.6%] and 211 females [99.1%]) and injury location (n = 332 [87.4%]; 143 males [85.6%] and 189 females [88.7%]). Zip Code Rural-Urban Commuting Area (RUCA) codes (2010) were used to determine whether decedents lived in nonmetropolitan versus metropolitan areas. RUCA codes measure daily commuting flows, population density, and urbanization levels to classify sub-county level geographic areas. Victim residential Zip codes were dichotomized as “metro” (RUCA codes 1–3) and “nonmetro” (RUCA codes 4–10). Descriptions of the RUCA classification codes 1–10 are available at https://www.ers.usda.gov/data-products/rural-urban-commuting-area-codes/documentation. ^§§^ Other method includes (in descending order): motor vehicles (e.g., buses, motorcycles, other transport vehicles), fire/burns, poisoning, fall, and other (single method). ^¶¶^ Other location includes (in descending order): hotel/motel, motor vehicle, parking lot/public garage/public transport, park/playground/sports or athletic area, commercial/retail area, synagogue/church/temple, hospital or medical facility, office building, and other unspecified location.

Nearly half of AI/AN victims of IPV-related homicide lived or were killed in metropolitan areas (44.4% and 46.1%, respectively) (Table 4). A firearm was the most common mechanism of IPV-related homicide injury overall (42.6%) and for both AI/AN males and females (46.1% and 39.9%, respectively). More IPV-related homicides of AI/AN males than females were perpetrated using a sharp instrument (33.5% versus 16.9%), and more IPV-related homicides of AI/AN females than males were perpetrated using personal weapons (e.g., hands, feet, or fists) (13.6% versus 7.2%); blunt instrument (11.7% versus 6.0%); and hanging, strangulation, or suffocation (9.9% versus 2.4%). Most (72.6%) AI/AN victims of IPV-related homicide were killed in a house or apartment. More AI/AN females than males were killed in a natural area (e.g., field or river) (6.1% versus 2.4%), whereas more AI/AN males than females were killed on a street or highway (12.0% versus 6.6%). More AI/AN females than males were killed in their own home (58.2% versus 47.3%).

### Characteristics of IPV-Related Homicide Suspects

A suspect was identified in nearly all (98.9%) IPV-related AI/AN homicides (Table 5). The age, sex, and race/ethnicity of the suspect were known in 80.6%, 98.9%, and 83.2% of cases, respectively. One fourth (25.5%) of suspects were aged 25–34 years. Most (95.7%) IPV-related homicides of AI/AN females were perpetrated by male suspects compared with 57.6% of IPV-related homicides of AI/AN males. More than one third (35.4%) of suspects were AI/AN, 47.9% were non-AI/AN, and the race/ethnicity of the remaining 16.8% was unknown. Almost two thirds (65.0%) of non-AI/AN suspects were non-Hispanic White, 16.7% were non-Hispanic Black, 12.8% were Hispanic (any race except AI/AN), and 5.6% were non-Hispanic Asian or NHOPI. The victim’s relationship to the suspect for a higher proportion of AI/AN female victims than male victims was a current intimate partner (72.0% versus 37.6%), former intimate partner (10.0% versus 4.2%), or intimate partner but the status of the relationship was unclear (i.e., unknown whether a current or former intimate partner; 5.2% versus 1.2%), whereas a higher proportion of AI/AN male victims than female victims were corollary victims of IPV-related homicide (51.5% versus 10.9%). In particular, a higher percentage of suspects for AI/AN male victims than female victims were an acquaintance or friend (21.8% versus 3.3%), a person known to the victim but the exact nature of the relationship was unclear (11.5% versus 3.3%), or other nonintimate partner (18.2% versus 4.3%).

**TABLE 5:** Number and percentage of American Indian/Alaska Native intimate partner violence-related homicides,* by victim’s sex, selected characteristics of homicide suspects, and the victim’s relationship to the suspect — National Violent Death Reporting System,† 2003–2018

Characteristic	Male (n = 165) No. (%)^§^	Female (n = 211) No. (%)	Total (n = 376) No. (%)
**Suspect age group (yrs)**			
<18	5 (3.0)	2 (<1.0)	**7 (1.9)**
18–24	19 (11.5)	31 (14.7)	**50 (13.3)**
25–34	49 (29.7)	47 (22.3)	**96 (25.5)**
35–44	25 (15.2)	51 (24.2)	**76 (20.2)**
45–54	21 (12.7)	33 (15.6)	**54 (14.4)**
55–64	3 (1.8)	11 (5.2)	**14 (3.7)**
≥65	2 (1.2)	4 (1.9)	**6 (1.6)**
Unknown	41 (24.8)	32 (15.2)	**73 (19.4)**
**Suspect sex**			
Male	95 (57.6)	202 (95.7)	**297 (79.0)**
Female	67 (40.6)	8 (3.8)	**75 (19.9)**
Unknown	3 (1.8)	1 (<1.0)	**4 (1.1)**
**Suspect race/ethnicity**			
AI/AN	63 (38.2)	70 (33.2)	**133 (35.4)**
Not AI/AN	69 (41.8)	111 (52.6)	**180 (47.9)**
White, non-Hispanic	55 (79.7)	62 (55.9)	**117 (65.0)**
Black, non-Hispanic	6 (8.7)	24 (21.6)	**30 (16.7)**
Hispanic or Latino, any race except AI/AN	7 (10.1)	16 (14.4)	**23 (12.8)**
Asian/Pacific Islander, non-Hispanic	1 (1.4)	9 (8.1)	**10 (5.6)**
Unknown, non-Hispanic	33 (20.0)	30 (14.2)	**63 (16.8)**
**Victim’s relationship to suspect^¶^**			
Intimate partner	71 (43.0)	184 (87.2)	**255 (67.8)**
Current intimate partner	62 (37.6)	152 (72.0)	**214 (56.9)**
Former intimate partner	7 (4.2)	21 (10.0)	**28 (7.4)**
Intimate partner, unknown whether current or former	2 (1.2)	11 (5.2)	**13 (3.5)**
Nonintimate partner (i.e., corollary victims)	85 (51.5)	23 (10.9)	**108 (28.7)**
Acquaintance/friend	36 (21.8)	7 (3.3)	**43 (11.4)**
Other person, known to victim	19 (11.5)	7 (3.3)	**26 (6.9)**
Other relationship**	30 (18.2)	9 (4.3)	**39 (10.4)**
Unknown	9 (5.5)	4 (1.9)	**13 (3.5)**

**Abbreviation:** AI/AN = American Indian Alaska Native.

*Includes victims killed by an intimate partner (e.g., current, former, or unspecified spouse, boyfriend, or girlfriend), victims killed during an intimate partner violence-related homicide who were not the intimate partner of the suspect (e.g., family, friends, and others who might have intervened in intimate partner violence, such as first responders or bystanders), and homicides precipitated by jealousy or distress over an intimate partner’s relationship or suspected relationship with another person. ^†^ NVDRS data have been collected in Maryland, Massachusetts, New Jersey, Oregon, South Carolina, and Virginia since 2003; Alaska, Colorado, Georgia, North Carolina, Oklahoma, Rhode Island, and Wisconsin since 2004; Kentucky, New Mexico, and Utah since 2005; Ohio since 2011; Michigan since 2014; Arizona, Connecticut, Hawaii, Illinois, Indiana, Iowa, Kansas, Maine, Minnesota, New Hampshire, New York, Pennsylvania, Vermont, and Washington since 2015; and Alabama, California, Delaware, District of Columbia, Louisiana, Missouri, Nebraska, Nevada, and West Virginia since 2017. Three states reported data on a subset of counties that represented at least 80% of violent deaths in their state during 2016–2018 (Illinois and Pennsylvania) and 2016–2017 (Washington). California reported 2017 data from four counties (Los Angeles, Sacramento, Shasta, and Siskiyou) and 2018 data from 21 counties (Amador, Butte, Fresno, Humboldt, Imperial, Kern, Kings, Lake, Los Angeles, Marin, Mono, Placer, Sacramento, San Benito, San Diego, San Francisco, San Mateo, Shasta, Siskiyou, Ventura, Yolo). Hawaii provided data for 2015–2016 because of lack of complete data in other years. ^§^ Percentages might not total 100% due to rounding and are based on the number of intimate partner violence-related homicide decedents with a known suspect (n = 376 [98.9%]; 165 males [98.8%] and 211 females [99.1%]). ^¶^ The following sentence can be used as a general guide for interpreting victim-suspect relationship: “The victim is the ____________ of the suspect.” For example, when a parent kills a child, the relationship is “Child” not “Parent” (“The victim is the child of the suspect”). The sentence is a general guide and some relationships might not be captured by this sentence (e.g., other person known to victim or victim was a law enforcement officer killed in the line of duty). ** Other relationship includes (in descending order): other relative, child, child of suspect's boyfriend/girlfriend (e.g., child killed by mother's boyfriend), stranger, parent, and intimate partner of suspect's parent (e.g., teenager kills his mother’s boyfriend).

### Circumstances of IPV-Related Homicide

All but two of the 380 IPV-related homicides (99.5%) had known circumstance information (Table 6). Nearly half (45.8%) of IPV-related homicides were precipitated by an argument or conflict. Jealousy over an actual or perceived relationship precipitated more IPV-related homicides of AI/AN males than females (30.5% versus 13.7%). Jealousy also was more common in IPV-related homicides in which the suspect killed a nonintimate partner (i.e., corollary victim) versus an intimate partner, particularly for male victims (45.8% versus 9.9% for male victims and 17.2% versus 13.0% for female victims, respectively). A crime precipitated 15.3% of IPV-related homicides (i.e., the homicide occurred as the result of another serious crime), and the crime was in progress at the time of the fatal injury in 56.9% of these incidents. The type of crime most frequently precipitating IPV-related homicides of AI/AN males was assault or homicide (68.8%), burglary (12.5%), rape or sexual assault (9.4%), and robbery (9.4%) and for females was assault or homicide (46.2%), rape or sexual assault (34.6%), arson (7.7%), and burglary (7.7%). More IPV-related homicides of AI/AN males than females were precipitated by a physical fight between two persons (34.2% versus 11.2%). More AI/AN female than male victims of IPV-related homicide were known to have experienced interpersonal violence during the month preceding their death (15.9% versus 4.5%). IPV-related homicide victims were reported to have had a problem with substance use, alcohol, or both in 25.9% of cases; 16.5% of victims had a noted problem with substances other than alcohol and 16.1% with alcohol. Although less common, 4.3% of IPV-related homicide victims were reported to have a current diagnosis of a mental health problem, and 2.4% with a current depressed mood at the time of death; 1.6% of victims were reported to currently be receiving mental health treatment.

**TABLE 6:** Number and percentage of American Indian/Alaska Native intimate partner violence-related homicides,* by victim’s sex and circumstance — National Violent Death Reporting System,^†^ 2003–2018

Circumstance^§^	Male No. (%)^¶^	Female No. (%)	Total No. (%)
**Total homicides**	**167**	**213**	**380**
**Homicides with known circumstances (% of total)**	**167 (100)**	**211 (99.1)**	**378 (99.5)**
Mental health/Substance use**			
Substance use problem (excluding alcohol)	20 (18.2)	22 (15.2)	**42 (16.5)**
Alcohol problem	19 (17.3)	22 (15.2)	**41 (16.1)**
Current diagnosed mental health problem	2 (1.8)	9 (6.2)	**11 (4.3)**
History of ever being treated for a mental health problem	1 (<1.0)	7 (4.8)	**8 (3.1)**
Current depressed mood	2 (1.8)	4 (2.8)	**6 (2.4)**
Current mental health treatment	0 (0.0)	4 (2.8)	**4 (1.6)**
Other addiction (e.g., gambling or sex)	0 (0.0)	0 (0.0)	**0 (0.0)**
**Interpersonal**			
Jealousy	51 (30.5)	29 (13.7)	**80 (21.2)**
Victim of interpersonal violence during past month	5 (4.5)	23 (15.9)	**28 (11.0)**
Other relationship problem (nonintimate)	10 (6.0)	6 (2.8)	**16 (4.2)**
Perpetrator of interpersonal violence during past month	8 (7.3)	2 (1.4)	**10 (3.9)**
Family relationship problem**	7 (6.4)	1 (<1.0)	**8 (3.1)**
**Life stressor**			
Argument or conflict	82 (49.1)	91 (43.1)	**173 (45.8)**
Physical fight (two persons, not a brawl)^††^	26 (34.2)	11 (11.2)	**37 (21.3)**
Crisis during previous or upcoming 2 weeks	9 (5.4)	16 (7.6)	**25 (6.6)**
History of child abuse/neglect	0 (0.0)	0 (0.0)	**0 (0.0)**
**Crime and criminal activity**			
Precipitated by another crime	32 (19.2)	26 (12.3)	**58 (15.3)**
Crime in progress^§§^	16 (50.0)	17 (65.4)	**33 (56.9)**
Drug involvement	11 (6.6)	8 (3.8)	**19 (5.0)**
Gang related	5 (3.0)	2 (<1.0)	**7 (1.9)**
Terrorist attack	0 (0.0)	0 (0.0)	**0 (0.0)**
**Homicide event**			
Caretaker abuse/neglect led to death^††^	1 (1.3)	7 (7.1)	**8 (4.6)**
Justifiable self-defense	12 (7.2)	0 (0.0)	**12 (3.2)**
Victim used a weapon	9 (5.4)	0 (0.0)	**9 (2.4)**
Mentally ill suspect^¶¶^	3 (1.8)	4 (1.9)	**7 (1.9)**
Brawl	5 (3.0)	1 (<1.0)	**6 (1.6)**
Victim was an intervener assisting a crime victim	2 (1.2)	2 (<1.0)	**4 (1.1)**
Victim was a bystander	1 (<1.0)	2 (<1.0)	**3 (<1.0)**
Drive-by shooting**	2 (1.8)	0 (0.0)	**2 (<1.0)**
Walk-by assault^††^	0 (0.0)	1 (1.0)	**1 (<1.0)**
Stalking^††^	0 (0.0)	1 (1.0)	**1 (<1.0)**
Victim was a police officer on duty	0 (0.0)	0 (0.0)	**0 (0.0)**
Mercy killing	0 (0.0)	0 (0.0)	**0 (0.0)**
Hate crime	0 (0.0)	0 (0.0)	**0 (0.0)**
Random violence**	0 (0.0)	0 (0.0)	**0 (0.0)**
Prostitution^††^	0 (0.0)	0 (0.0)	**0 (0.0)**

* Includes victims killed by an intimate partner (e.g., current, former, or unspecified spouse, boyfriend, or girlfriend), victims killed during an intimate partner violence-related homicide who were not the intimate partner of the suspect (e.g., family, friends, and others who might have intervened in intimate partner violence, such as first responders or bystanders), and homicides precipitated by jealousy or distress over an intimate partner’s relationship or suspected relationship with another person. ^†^ NVDRS data have been collected in Maryland, Massachusetts, New Jersey, Oregon, South Carolina, and Virginia since 2003; Alaska, Colorado, Georgia, North Carolina, Oklahoma, Rhode Island, and Wisconsin since 2004; Kentucky, New Mexico, and Utah since 2005; Ohio since 2011; Michigan since 2014; Arizona, Connecticut, Hawaii, Illinois, Indiana, Iowa, Kansas, Maine, Minnesota, New Hampshire, New York, Pennsylvania, Vermont, and Washington since 2015; and Alabama, California, Delaware, District of Columbia, Louisiana, Missouri, Nebraska, Nevada, and West Virginia since 2017. Three states reported data on a subset of counties that represented at least 80% of violent deaths in their state during 2016–2018 (Illinois and Pennsylvania) and 2016–2017 (Washington). California reported 2017 data from four counties (Los Angeles, Sacramento, Shasta, and Siskiyou) and 2018 data from 21 counties (Amador, Butte, Fresno, Humboldt, Imperial, Kern, Kings, Lake, Los Angeles, Marin, Mono, Placer, Sacramento, San Benito, San Diego, San Francisco, San Mateo, Shasta, Siskiyou, Ventura, Yolo). Hawaii provided data only for 2015–2016 because of lack of complete data in other years. ^§^ Includes intimate partner violence-related homicides with one or more circumstances. Total numbers do not equal the sums of the columns because more than one circumstance could have been present per decedent. ^¶^ Percentage is based on the number of intimate partner violence–related homicides with known circumstances. ** Variable collected for intimate partner violence-related homicides since 2009. Denominator is intimate partner violence-related homicides with known circumstances during 2009-2018 (n = 255; 110 males and 145 females). ^††^ Variable collected for homicides since 2013. Denominator is homicides with known circumstances during 2013–2018 (n = 174; 76 males and 98 females). ^§§^ Denominator includes those decedents involved in an incident that was precipitated by another crime. ^¶¶^ Percentage is based on the number of intimate partner violence-related homicide decedents with a known suspect (n = 376 [98.9%]; 165 males [98.8%] and 211 females [99.1%]).

## Discussion

MMIP is an issue of urgent concern as indicated by the formation of the Presidential Task Force on Missing and Murdered American Indians and Alaska Natives and the passage and signing of Savanna’s Act and the Not Invisible Act (*1*–*3*). NVDRS data can be used to characterize and monitor AI/AN homicides and identify effective and early intervention strategies for preventing such deaths. This report presents the most comprehensive analysis to date using NVDRS data to examine homicides of AI/ANs and the circumstances surrounding these deaths. Consistent with the homicide rates for other racial/ethnic groups (*25*), the homicide rate for AI/AN males was higher than the rate for females. A firearm was used in nearly half of AI/AN homicides, and one fourth of homicides were precipitated by another serious crime (e.g., robbery and sexual assault). More than half of AI/AN homicides occurred in a residence, and one third occurred in the victim’s home. Interpersonal conflict was a predominant circumstance, with nearly half of AI/AN homicides precipitated by an argument; for female victims, 45.0% were precipitated by IPV. Findings from this report can be used to guide the work of the Presidential Task Force on Missing and Murdered American Indians and Alaska Natives and others to address AI/AN homicide (*1*).

NVDRS programs have used their local Violent Death Reporting System (VDRS) data to examine AI/AN homicides. For example, Arizona VDRS examined AI/AN homicides in their state during 2015–2017 (3.1% of all homicides) and found that the homicide rates for AI/ANs were more than double the rates for non-AI/ANs among both males (20.0 versus 8.7 per 100,000 population) and females (5.8 versus 8.7) (*29*). Further examination revealed that the characteristics and circumstances of AI/AN victims and homicides differed from those of non-AI/ANs. Notably, AI/AN homicide victims had completed substantially less education than non-AI/AN victims; 9% of AI/AN male victims and few, if any, AI/AN female victims had earned, at minimum, some college credit or degree compared with 22.8% of non-AI/AN male victims and 44.8% of non-AI/AN female victims. AI/AN male and female homicide victims were more likely than non-AI/AN male and female victims to never have been married (for males, 88.2% versus 59.7%; for females, 63.2% versus 34.6%, respectively). Female victims, both AI/AN (31.3%) and non-AI/AN (45.0%), were at significantly greater risk for homicide by current or former intimate partners than their male counterparts (fewer than 5 victims and 3.9%, respectively). AI/AN male and female homicide victims were significantly less likely than non-AI/AN victims to have been killed with a firearm (for males, 42.9% versus 73.9%; for females, 40.9% versus 65.4%, respectively). Oklahoma VDRS examined AI/AN homicides in their state during 2004–2008 and found that the overall homicide rate among AI/ANs was 20% higher than the overall rate among non-AI/ANs, and the homicide rate among AI/ANs aged 25–44 years was nearly double that of non-AI/ANs of the same age group (*30*). Arguments and IPV were the leading circumstances of homicide among AI/AN victims (49% and 22%, respectively). These details from examination of local VDRS data increase the knowledge base about the circumstances associated with AI/AN homicide and can assist local public health authorities and their partners in developing and guiding effective, data-driven approaches to violence prevention.

From 2000 to 2010, the AI/AN population increased almost twice as fast as the total U.S. population (*31*), with the percentage of AI/AN persons aged <18 years higher than the percentage of those aged <18 years for the total U.S. population (29.0% versus 21.9%) (*32*). AI/AN youths, particularly young adult males, experience disproportionate rates of violent injury and homicide (*7*,*22*,*25*). Socioeconomic factors (e.g., lack of economic opportunity, income inequality, and poverty) contribute to violence and have been associated with higher rates of homicide (*33*). According to the 2000 U.S. Census, more AI/ANs lived below the federal poverty level than those from all other racial/ethnic groups (25.7% versus 12.4%) (*34*). Thirteen percent of AI/AN males aged ≥16 years were unemployed compared with 5.7% of males aged ≥16 years in all other racial/ethnic groups; unemployment for AI/AN females aged ≥16 years was 11.7% compared with 5.8% of females in all other racial/ethnic groups (*34*). In 2019, AI/ANs were less likely to have high school diplomas or higher-level education than non-Hispanic Whites (84.4% versus 93.3%) (*17*). To improve outcomes and reduce violence in AI/AN communities, exploring economic opportunity and addressing other social determinants of health, such as education, should be considered (*35*).

Several findings are relevant to the understanding of AI/AN homicides and have implications for violence prevention efforts. Approximately half of AI/AN homicide victims lived or were killed in metropolitan areas. Misperceptions exist about where AI/ANs live and how they access resources (*20*). Although more than half of AI/ANs lived in rural areas in 1970, approximately 70% now live in urban areas, which affects their access to health care and other services (*36*,*37*). AI/AN populations face persistent disparities in health and health care, including high uninsured rates, significant barriers to obtaining care, and poor health status (*38*). AI/ANs who are members of federally recognized tribes and some descendants have legal rights to and are eligible for health care from the federal government through the Indian Health Service (IHS) (*34*,*39*). However, IHS has facilities in only 35 states, primarily on or near rural reservation lands and in few cities with large AI/AN populations (*40*), and IHS provides health services to only 2.6 million of the 6.9 million persons reporting AI/AN ancestry in the United States (*16*,*17*). In addition, the overall IHS budget meets just over half of the demonstrated health care needs of the eligible AI/AN population (*41*). IHS focuses on providing primary health care at no cost to patients, with limited specialty care and inpatient services, which might not meet the needs of victims of violence or primary prevention programs working to address violence as a public health problem (*40*,*41*). AI/ANs in rural areas might experience further challenges related to social isolation and cultural and transportation barriers (*42*) that hinder their ability to access health care and other resources (*43*). Thus, violence prevention efforts in health care–based settings, such as IHS, might be limited, and community approaches considering the needs of AI/ANs in urban versus rural settings are needed to reduce violence and homicide in these communities.

Interpersonal conflicts, such as arguments, IPV, and problems with family members and other nonintimate partners, were prominent circumstances of AI/AN homicides, most of which were perpetrated by male suspects and suspects the victim knew. AI/AN homicide victims are commonly perceived to be killed by non-AI/AN suspects, particularly White males (*44*). Information about perpetrators of violence against AI/ANs are frequently based on a 1999 U.S. Department of Justice report in which 60% of American Indian victims of violent crime described a White perpetrator (*44*). However, the findings in this report indicate that nearly one third of AI/AN victims were killed by AI/AN suspects and approximately 40% were killed by a racially diverse group of non-AI/AN suspects. Further research is needed to understand the interpersonal conflicts and racial/ethnic dynamics between victims and suspects that contribute to AI/AN homicides.

### IPV-Related Homicide

The results of this study provide further evidence that violence against AI/AN women is an issue of urgent concern (*43*). IPV was a contributing factor in nearly half of the homicides of AI/AN females. Rape or sexual assault occurred in nearly one third of IPV-related homicides precipitated by another serious crime. Data from the National Intimate Partner and Sexual Violence Survey and National Crime Victimization Survey indicate that AI/AN women experience higher rates of rape and sexual assault, physical assault, and stalking than women of other racial/ethnic groups (*13*,*43*). However, similar to women of other racial/ethnic groups, AI/AN women are likely to be victimized by someone they know (*43*). The findings in this report indicate that approximately 40% of female victims were killed by a current or former intimate partner and 12% by an acquaintance or friend. Results also indicate that leaving an intimate partner relationship remains dangerous for some victims (*45*). Ten percent of AI/AN female victims and 4.2% of male victims were killed by a former intimate partner.

The findings also indicate that some AI/ANs were corollary victims of IPV-related homicide, particularly among male victims (*46*). Such victims are those who were affected by IPV but were not the intimate partners themselves. These can include bystanders to an IPV incident, children of the IPV victim, family members or friends assisting the IPV victim, or persons involved in a true or perceived romantic relationship with the IPV victim (*46*). A large proportion of female victims of IPV-related homicide were killed by their current or former intimate partners. In contrast, approximately half of the male victims killed during an IPV-related incident were corollary victims. This finding indicates that many AI/AN male victims of IPV-related homicide might reflect a specific category of corollary victims, referred to as “other intimate partner involvement” (*46*). These are victims who were connected to the suspect through a mutual intimate partner, either currently or in the past, such as new boyfriends and partners killed by a former partner (*46*). This is also supported by findings related to jealousy over an actual or perceived relationship with a romantic rival. Nearly one third of IPV-related homicides involving male victims were precipitated by jealousy compared with approximately 14% of female victims. These findings indicate that the impact of IPV can extend beyond the couple involved, and that IPV prevention and intervention strategies that consider family, friends, and others who might be exposed to IPV can be most helpful in preventing IPV-related homicide.

The IPV-related homicides identified in this report, particularly among women, have several implications for the criminal justice system. However, AI/AN women experiencing IPV face unique challenges when seeking resources. Complicated jurisdictional issues might lead to inadequate or delayed responses by investigative authorities, and legal constraints on tribal sovereignty might limit tribal authority to prosecute offenders (*43*). Further, indigenous views of law differ from the U.S. legal system (*43*). Traditional AI/AN criminal justice systems emphasize communal values that restore harmony within the community (*47*,*48*), whereas the U.S. legal system typically emphasizes deterrence in the form of punishment and focuses on individual cases (*43*). This dissonance can create a cultural barrier that impedes healing and resolution. AI/AN women living on tribal lands might experience additional challenges of social isolation, inadequate access to resources, and cultural barriers when seeking assistance outside their community (*43*).

The Violence Against Women Act (VAWA) and its reauthorizations provided legislation for protecting female victims of violence and dedicated funding to combat and respond to violence against women (*49*,*50*). VAWA included initiatives to strengthen law enforcement in preventing violence against women; allocated funds to expedite the processing of rape kits and other victim services; and authorized the administering of grants to several programs designed to reduce domestic violence, dating violence, sexual assault, and stalking (*49*,*50*). VAWA’s reauthorization in 2013 addressed tribal sovereignty and jurisdictional issues related to violence against AI/ANs occurring on tribal lands. In particular, VAWA’s reauthorization in 2013 recognized the inherent ability of tribes to prosecute non-AI/AN domestic violence and stalking perpetrators and violators of orders of protection after certain criteria were met by tribes and the alleged perpetrators (*50*). In 2014, the Pascua Yaqui Tribe became the first tribe to prosecute a non-AI/AN domestic violence perpetrator in Pascua Yaqui Tribal Court (*51*). Tribal sovereignty and jurisdictional issues related to violence against AI/ANs and cross-sectoral collaborations (e.g., among public health professionals, law enforcement, and direct service providers) remain key focus areas for those working to address AI/AN homicide.

### Implications for Prevention

Violence prevention efforts might have greater impact if they include trauma-informed strategies, provide accessible behavioral health services, and use strengths-based approaches to promote resilience and other protective factors that are inherent in AI/AN communities (*21*). AI/AN populations possess community and cultural assets that protect against violence, such as community mindedness, connection to tribal leaders and elders, participation in tribal ceremonies, and spirituality (*21*,*52*,*53*). Native teachings and traditions help persons develop a sense of identity, and generational sharing of knowledge through songs and storytelling contribute to a sense of connectedness and community resilience (*52*,*53*). Such cultural practices are protective against violence among AI/AN youths (*52*) and can be integrated into violence prevention strategies in AI/AN communities.

CDC developed technical packages that summarize the best available evidence for preventing different types of violence (*54*). One critical strategy for preventing interpersonal violence is to teach safe and healthy relationship skills through social-emotional learning programs for youth (*55*). For example, “Safe Dates,” a school-based dating violence prevention program, has helped reduce teen dating violence among racial/ethnic minority adolescents (*56*) and could be adapted for use with AI/AN youths. “Fourth R: Strategies for Healthy Teen Relationships*”* (https://youthrelationships.org) is another evidence-based, social-emotional learning program that has been successfully adapted and implemented in AI/AN communities (*57*). Tribal communities also have developed similar programs that leverage their unique cultural and community assets. “Discovery Dating*”* is a strengths-based, healthy relationships curriculum rooted in Native American values that increased feelings of personal agency among Native American middle school youths (*58*) and reduced rates of teen pregnancy (*59*). Further research is needed to evaluate specific impacts on violence outcomes; however, programs like “Discovery Dating*”* represent culturally relevant interventions that can be tailored for tribal communities and are consistent with strategies identified in CDC’s technical packages.

Community-led action and attention on missing and murdered indigenous women and girls have led discussions and policy actions around AI/AN homicide (*1*). However, homicides of AI/AN males, particularly among youths and young adults, contribute to many AI/AN homicides. AI/AN communities can strengthen, evaluate, and adapt existing programs created specifically to address violence among AI/AN males. For example, “Boys Run I toowú klatseen*”* (https://boysrun.org) aims to build healthy relationships by promoting traditional tribal values and equitable gender norms among third- and fifth-grade Alaska Native boys (*60*). Multnomah County in Oregon partners with the Native American Youth Association to implement a culturally adapted version of “Coaching Boys Into Men” (https://www.coachescorner.org), an evidence-based program engaging male youths that builds knowledge and prosocial attitudes about healthy masculinity, healthy relationships, nonviolent problem solving, and being an active bystander (*61*).

This report identified circumstances of IPV-related AI/AN homicides that could guide other evidence-based approaches, such as bystander programs and IPV lethality risk assessments, that can be adapted for use in AI/AN communities. Approximately 15% of AI/AN female victims and 4.5% of AI/AN male victims of IPV-related homicide experienced some form of violence in the preceding month, which could have provided opportunities to intervene and prevented escalating violence that resulted in the homicide. Bystander programs such as “Green Dot*”* teach participants how to recognize situations or behaviors that might become violent and how to intervene safely and effectively in IPV and other violence (*62*). IPV lethality risk assessments are used by law enforcement officers responding to the scene of a domestic violence incident to facilitate immediate safety planning and to connect IPV victims with other services, such as crisis intervention and counseling, housing, medical and legal advocacy, and other community resources (*63*). These assessments help identify victims at risk for future violence and might have promise in preventing intimate partner homicide (*63*).

Other strategies to prevent violence include reducing exposure to community-level risks and strengthening economic supports. Community- and societal-level programs, such as “Crime Prevention Through Environmental Design” (*64*), business improvement districts (*65*,*66*), and alcohol-related policies, promote protective community environments and reduce the risk for violence (*33*). The earned income tax credit enhances household financial security by raising family income while incentivizing work and counterbalancing the costs of child rearing, which in turn improve home environments and encourage healthy development (*67*,*68*). A quasi-experimental study of the effects of cash supplements from casino revenue on an Eastern Cherokee reservation found that an additional $4,000 per year for the poorest American Indian households increased educational attainment by 1 year at age 21 years and reduced the incidence of criminality by 22% at ages 16 and 17 years (*69*). The strategies described indicate that a multidisciplinary approach that cuts across different sectors and addresses the unique values and cultural needs of AI/AN communities might be warranted to reduce violence and AI/AN homicide.

## Limitations

The findings in this report are subject to at least six limitations. First, racial misclassification of AI/AN decedents on death certificates and in other investigative reports is a known concern (*22*,*70*) and might have contributed to an underestimate of AI/AN homicides. Racial misclassification is often caused by inaccurate information submitted by health care personnel or by death certifiers basing classification on appearance or surname alone (*22*). This is compounded by jurisdictional issues in death investigations that occur on tribal lands because information from these investigative reports might not be captured in federal systems, such as NVDRS, because of a lack of routine data sharing (*43*). Each tribe has inherent legal and political authority to govern itself (i.e., tribal sovereignty) (*19*) and might not interact with the investigative and public health entities that typically provide data for NVDRS.

Second, NVDRS does not collect information on tribal affiliation, residence on reservations, or federally recognized tribally governed lands or whether homicides occurred on tribal lands. Linking with tribal registries or other tribal data sources would enable more reliable collection of data. Third, the rates and characteristics of homicides of different tribes could not be assessed; therefore, the findings in this report might not be generalizable across all AI/AN communities. Future studies might consider examining differences across tribes, given the diverse cultural practices and beliefs that might affect health outcomes in AI/AN communities (*36*). Fourth, NVDRS data are not nationally representative, and not all states joined the system at the same time (*25*). Therefore, it was not possible to use NVDRS data to report regional disparities in homicide rates, which have been demonstrated by other studies of AI/AN homicide (*22*).

Fifth, the availability and completeness of the data are dependent on successful partnerships among local VDRS programs and their partners in vital records, medical examiner and coroner offices, and law enforcement (*24*). NVDRS data might be limited or incomplete for areas in which these data-sharing relations are not fully developed. Further, some state VDRS programs do not receive detailed law enforcement reports until cases are adjudicated (*24*), which might result in an underestimate of circumstance information. Child abuse and neglect (*8*,*11*,*71*) and human trafficking (*72*–*74*) are known issues of concern in AI/AN populations but were likely underreported in NVDRS. In addition, medical, mental health, and substance use information are not captured directly from medical records and might not be systematically collected for victims unless they were directly related to the homicide. Therefore, the completeness and accuracy of this information is limited.

Finally, NVDRS collects limited information about suspects. Data on certain suspect characteristics, such as alcohol and other substance use, were recently added to the system but were not available for this report (*26*).

## Conclusion

This report presents a detailed examination of the circumstances surrounding AI/AN homicides and highlights the role of IPV in these deaths. NVDRS data can be used to characterize and monitor AI/AN homicides and identify effective and early intervention strategies for preventing such deaths. Future studies of NVDRS data could show unique risk factors for homicide in the AI/AN population, changing trends, and regional variations in AI/AN homicide. An integrated primary prevention and health promotion response that coordinates across tribes, the federal government, public health and health services sectors, the criminal justice system, and victim services is important in addressing violence among AI/AN populations. To support prevention efforts and to fully address the complexities of MMIP and, particularly, AI/AN homicide, AI/AN communities might need collective healing of historic and intergenerational traumas and resolution of structural inequities (*20*). AI/AN communities are rich in native knowledge, teachings, and protective factors, which can be used to further violence prevention efforts (*20*). When possible, violence prevention efforts should include community-developed, culturally relevant, and evidence-based strategies; incorporate traditional native knowledge and solutions; and consider the influence of historical and larger societal factors that might increase the likelihood of violence in AI/AN communities.

## Footnotes

* Other terms commonly used might include but are not limited to Missing and Murdered Indigenous Women, Missing and Murdered Indigenous Women, Girls, and Two-Spirit People.

† Information on race and ethnicity are recorded as separate items in NVDRS consistent with U.S. Department of Health and Human Services (HHS) and Office of Management and Budget standards for race/ethnicity categorization. HHS guidance on race/ethnicity is available at https://aspe.hhs.gov/basic-report/hhs-implementation-guidance-data-collection-standards-race-ethnicity-sex-primary-language-and-disability-status.

§ Zip Code Rural-Urban Commuting Area (RUCA) codes (2010) were used to determine whether decedents lived in nonmetropolitan versus metropolitan areas. RUCA codes measure daily commuting flows, population density, and urbanization levels to classify subcounty level geographic areas. Victim residential Zip codes and Zip codes where injury occurred were dichotomized as “metro” (RUCA codes 1–3) and “nonmetro” (RUCA codes 4–10). Descriptions of the RUCA classification codes 1–10 are available at https://www.ers.usda.gov/data-products/rural-urban-commuting-area-codes/documentation.

## Appendix

### Circumstances preceding homicide — National Violent Death Reporting System

**Mental health/substance use**

- Current depressed mood: decedent was perceived by self or others to be feeling depressed at the time of death.
- Current diagnosed mental health problem: decedent was identified as having a mental health disorder or syndrome listed in the *Diagnostic and Statistical Manual, Fifth Version* (DSM-5), with the exception of alcohol and other substance dependence (these are captured in separate variables).
- Current mental health treatment: decedent was receiving mental health treatment as evidenced by a current prescription for a psychotropic medication, visit or visits to a mental health professional, or participation in a therapy group within the previous 2 months.
- History of ever being treated for mental health problem: decedent was identified as having ever received mental health treatment.
- Alcohol problem: decedent was perceived by self or others to have a problem with, or to be addicted to, alcohol.
- Substance use problem (excludes alcohol): decedent was perceived by self or others to have a problem with, or be addicted to, a substance other than alcohol.
- Other addiction: decedent was perceived by self or others to have an addiction other than to alcohol or other substance (e.g., gambling or sex).

**Interpersonal**

- Family relationship problem: decedent was experiencing problems with a family member, other than an intimate partner.
- Intimate partner violence–related: incident is related to conflict between current or former intimate partners; includes the death of an intimate partner or nonintimate partner (e.g., child, parent, friend, or law enforcement officer) killed in an incident that originated in a conflict between intimate partners.
- Jealousy (lovers’ triangle): jealousy or distress over an intimate partner’s relationship or suspected relationship with another person.
- Other relationship problem (nonintimate): decedent was experiencing problems with a friend or associate (other than an intimate partner or family member).
- Victim of interpersonal violence during previous month: decedent was the target of interpersonal violence during the past month.
- Perpetrator of interpersonal violence during previous month: decedent perpetrated interpersonal violence during the previous month.

**Life stressor**

- Argument or conflict: a specific argument or disagreement led to the victim’s death.
- Crisis during previous or upcoming 2 weeks: current crisis or acute precipitating event or events that either occurred during the previous 2 weeks or was impending in the following 2 weeks (e.g., a trial for a criminal offense begins the following week) and appeared to have contributed to the death. Crises typically are associated with specific circumstance variables (e.g., family relationship problem was a crisis).
- History of child abuse/neglect: as a child, decedent had history of physical, sexual, or psychological abuse; physical (including medical or dental), emotional, or educational neglect; exposure to a violent environment, or inadequate supervision by a caretaker.
- Physical fight (two persons, not a brawl): a physical fight between two persons that resulted in the death of the decedent, who was either involved in the fight, a bystander, or trying to stop the fight.

**Crime and criminal activity**

- Drug involvement: drug dealing, drug trade, or illicit drug use.
- Gang related: incident resulted from gang activity or gang rivalry; not used if the decedent was a gang member and the death did not appear to result from gang activity.
- Precipitated by another crime: incident occurred as the result of another serious crime.
- Nature of crime: the specific type of other crime that occurred during the incident. Examples include the following:
  - Arson: to unlawfully and intentionally damage, or attempt to damage, any building, real estate, or personal property by fire or incendiary device.
  - Assault or homicide: an unlawful fatal or nonfatal attack by one person upon another. To qualify as a serious crime, the assault should be an aggravated assault (one that involves bodily injury or threat with a deadly weapon).
  - Burglary: the unlawful entry into a building or other structure without the owner’s consent and with the intent to commit a felony or a theft.
  - Drug trade: the buying, selling, or passing of drugs from one person to another in exchange for goods or money.
  - Robbery: taking, or attempting to take, anything of value from another person or persons by force or threat of force or violence.
  - Rape or sexual assault: a sexual act that is committed or attempted by another person without freely given consent of the victim or against someone who is unable to consent or refuse.
- Crime in progress: another serious crime was in progress at the time of the incident.
- Terrorist attack: decedent was injured in a terrorist attack, leading to death.

**Homicide event**

- Brawl: mutual physical fight involving three or more persons.
- Caretaker abuse/neglect led to death: decedent was experiencing physical, sexual, or psychological abuse; physical (including medical or dental), emotional, or educational neglect; exposure to a violent environment; or inadequate supervision by a caretaker that led to death.
- Drive-by shooting: suspect drove near the decedent and fired a weapon while driving.
- Hate crime: decedent was selected intentionally because of decedent’s actual or perceived gender, religion, sexual orientation, race, ethnicity, or disability.
- Justifiable self-defense: decedent was killed by a law enforcement officer in the line of duty or by a civilian in legitimate self-defense or in defense of others.
- Mentally ill suspect: suspect’s attack on decedent was believed to be the direct result of a mental illness.
- Mercy killing: decedent wished to die because of a terminal or hopeless disease or condition, and documentation indicates that the decedent wanted to be killed.
- Prostitution: prostitution or related activity that includes prostitutes, pimps, clients, or others involved in such activity.
- Random violence: decedent was killed in a random act of violence (i.e., an act in which the suspect is not concerned with who is being harmed, just that someone is being harmed).
- Stalking: pattern of unwanted harassing or threatening tactics by either the decedent or suspect.
- Victim was a bystander: decedent was not the intended target in the incident (e.g., pedestrian walking past a gang fight).
- Victim was an intervener assisting a crime victim: decedent was attempting to assist a crime victim at the time of the incident (e.g., a child attempts to intervene and is killed while trying to assist a parent who is being assaulted).
- Victim was a police officer on duty: decedent was a law enforcement officer killed in the line of duty.
- Victim used a weapon: decedent used a weapon to attack or defend during the course of the incident.
- Walk-by assault: decedent was killed by a targeted attack (e.g., ambush) after which the suspect fled on foot.

## References

[1] Executive Order 13898 Establishing the Task Force on Missing and Murdered American Indians and Alaska Natives. Fed Regist 2019;84:66059–61.

[2] Savanna’s Act. S. 227, 116th Cong. (2020). https://www.congress.gov/116/plaws/publ165/PLAW-116publ165.pdf

[3] Not Invisible Act of 2019, S. 982, 116th Cong. (2020). https://www.congress.gov/116/plaws/publ166/PLAW-116publ166.pdf

[4] CDC. Web-based Injury Statistics Query and Reporting System (WISQARS). Atlanta, GA: US Department of Health and Human Services, CDC, National Center for Injury Prevention and Control; 2019. https://www.cdc.gov/injury/wisqars/index.html

[5] Perry SW. A BJS statistical profile, 1992–2002: American Indians and crime. Washington, DC: US Department of Justice, Office of Justice Programs; 2004. https://www.bjs.gov/content/pub/pdf/aic02.pdf

[6] Kenney MK, Singh GK. Adverse childhood experiences among American Indian/Alaska Native children: the 2011-2012 National Survey of Children’s Health. Scientifica (Cairo) 2016;2016:7424239. 10.1155/2016/7424239 27529052 PMC4977380

[7] Andreescu V, Overstreet SM. Violent victimization and violence perpetration among American Indian adolescents. J Interpers Violence 2020;886260520967313. 10.1177/0886260520967313 33092436

[8] Sapra KJ, Jubinski SM, Tanaka MF, Gershon RR. Family and partner interpersonal violence among American Indians/Alaska Natives. Inj Epidemiol 2014;1:7. 10.1186/2197-1714-1-7 27747668 PMC5005741

[9] Evans-Campbell T. Historical trauma in American Indian/Native Alaska communities: a multilevel framework for exploring impacts on individuals, families, and communities. J Interpers Violence 2008;23:316–38. 10.1177/0886260507312290 18245571

[10] Bombay A, Matheson K, Anisman H. The intergenerational effects of Indian residential schools: implications for the concept of historical trauma. Transcult Psychiatry 2014;51:320–38. 10.1177/1363461513503380 24065606 PMC4232330

[11] Brockie TN, Heinzelmann M, Gill J. A framework to examine the role of epigenetics in health disparities among Native Americans. Nurs Res Pract 2013;2013:410395. 10.1155/2013/410395 24386563 PMC3872279

[12] Gone JP, Hartmann WE, Pomerville A, Wendt DC, Klem SH, Burrage RL. The impact of historical trauma on health outcomes for indigenous populations in the USA and Canada: a systematic review. Am Psychol 2019;74:20–35. 10.1037/amp0000338 30652897

[13] Smith SG, Chen J, Basile KC, The National Intimate Partner and Sexual Violence Survey (NISVS): 2010–2012 State Report. Atlanta, GA: US Department of Health and Human Services, CDC; 2017. https://www.cdc.gov/violenceprevention/pdf/NISVS-StateReportBook.pdf

[14] Breiding MJ, Basile K, Smith SG, Black MC, Mahendra RR. Intimate partner violence surveillance: uniform definitions and recommended data elements, version 2.0. Atlanta, GA: US Department of Health and Human Services, CDC; 2015. https://www.cdc.gov/violenceprevention/pdf/intimatepartnerviolence.pdf

[15] Petrosky E, Blair JM, Betz CJ, Fowler KA, Jack SPD, Lyons BH. Racial and ethnic differences in homicides of adult women and the role of intimate partner violence—United States, 2003–2014. MMWR Morb Mortal Wkly Rep 2017;66:741–6. 10.15585/mmwr.mm6628a1 28727682 PMC5657947

[16] US Census Bureau. Facts for features: American Indian and Alaska Native Heritage Month: November 2020. Washington, DC: US Department of Commerce, Census Bureau; 2020. https://www.census.gov/newsroom/facts-for-features/2020/aian-month.html

[17] US Department of Health and Human Services. Profile: American Indian/Alaska Native. Rockville, MD: US Department of Health and Human Services, Office of Minority Health; 2021. https://www.minorityhealth.hhs.gov/omh/browse.aspx?lvl=3&lvlid=62

[18] Department of the Interior Indian Affairs. Bureau of Indian Affairs: mission statement. Washington, DC: US Department of the Interior; 2021. https://www.bia.gov/bia

[19] Worcester v. Georgia, 6 Pet. 515, 557 (1832). https://thorpe.law.ou.edu/treatises/cases/Worcester.PDF

[20] Satter DE, Kollar LM, Antone CL, American Indian and Alaska Native knowledge and public health for the primary prevention of Missing or Murdered Indigenous Persons. Department of Justice Journal of Federal Law and Practice 2021;69:149–88. 34734212 PMC8563020

[21] Substance Abuse and Mental Health Services Administration. The national tribal behavioral health agenda. Rockville, MD: US Department of Health and Human Services, Substance and Mental Health Services Administration; 2016. https://www.nihb.org/docs/12052016/FINAL%20TBHA%2012-4-16.pdf

[22] Herne MA, Maschino AC, Graham-Phillips AL. Homicide among American Indians/Alaska Natives, 1999-2009: implications for public health interventions. Public Health Rep 2016;131:597–604. 10.1177/0033354916662219 27453605 PMC4937122

[23] Connolly M, Gallagher M, Hodge F, Identification in a time of invisibility for American Indians and Alaska Natives in the United States. Stat J IAOS 2019;35:71–89. 10.3233/SJI-190495

[24] Blair JM, Fowler KA, Jack SP, Crosby AE. The National Violent Death Reporting System: overview and future directions. Inj Prev 2016;22(Suppl 1):i6–11. 10.1136/injuryprev-2015-041819 26718549 PMC6049659

[25] Petrosky E, Ertl A, Sheats KJ, Wilson R, Betz CJ, Blair JM. Surveillance for violent deaths—National Violent Death Reporting System, 34 states, four California counties, the District of Columbia, and Puerto Rico, 2017. MMWR Surveill Summ 2020;69(No. SS-8). 10.15585/mmwr.ss6908a1 33270620 PMC7713989

[26] CDC. National Violent Death Reporting System (NVDRS) coding manual version 5.4.1. Atlanta, GA: US Department of Health and Human Services, CDC; 2019. https://www.cdc.gov/violenceprevention/pdf/nvdrs/nvdrsCodingManual.pdf

[27] World Health Organization. International classification of diseases, tenth revision. Geneva, Switzerland: World Health Organization; 2007. https://icd.who.int/browse10/2019/en

[28] CDC. U.S. census populations with bridged race categories. Hyattsville, MD: US Department of Health and Human Services, CDC; 2019. https://www.cdc.gov/nchs/nvss/bridged_race.htm

[29] Arizona State University, Center for Violence Prevention and Community Safety. Homicides involving Native Americans, 2015–2017. Phoenix, AZ: Arizona Violent Death Reporting System; 2020. https://cvpcs.asu.edu/sites/default/files/content/products/homicide_native_american_final.pdf

[30] Kabore HJ. OK-VDRS Brief Report: violent deaths among Native Americans in Oklahoma. Oklahoma City, OK: Oklahoma State Department of Health; 2011. https://oklahoma.gov/content/dam/ok/en/health/health2/documents/okvdrs-brief-violent-death-among-native-americans.pdf

[31] Norris T, Vines PL, Hoeffel EM. The American Indian and Alaska Native population: 2010. Washington, DC: US Department of Commerce, US Census Bureau; 2010 https://www.census.gov/prod/cen2010/briefs/c2010br-10.pdf

[32] National Congress of American Indians. Indian country demographics. Washington, DC: National Congress of American Indians; 2001–2021. https://www.ncai.org/about-tribes/demographics

[33] David-Ferdon C, Vivolo-Kantor AM, Dahlberg LL, Marshall KJ, Rainford N, Hall JE. A comprehensive technical package for the prevention of youth violence and associated risk behaviors. Atlanta, GA: US Department of Health and Human Services, CDC; 2016. https://www.cdc.gov/violenceprevention/pdf/yv-technicalpackage.pdf

[34] Indian Health Service. Trends in Indian health: 2014 edition. Rockville, MD: US Department of Health and Human Services, Indian Health Service; 2015. https://www.ihs.gov/dps/index.cfm/publications/trends2014

[35] CDC. Social determinants of health: know what affects health. Atlanta, GA: US Department of Health and Human Services, CDC; 2021. https://www.cdc.gov/socialdeterminants

[36] Walters KL, Walls M, Dillard DA, Kaur JS. American Indian and Alaska Native research in the health sciences: critical considerations for the review of research applications. Bethesda, MD: National Institutes of Health, Tribal Health Research Office; 2019. https://dpcpsi.nih.gov/sites/default/files/Critical_Considerations_for_Reviewing_AIAN_Research_508.pdf

[37] National Archives and Records Administration. American Indian urban relocation. College Park, MD; National Archives; 2016. https://www.archives.gov/education/lessons/indian-relocation.html

[38] Office of the Assistant Secretary for Planning and Evaluation. Improving data capacity for American Indian/Alaska Native (AIAN) populations in federal health surveys. Washington, DC: US Department of Health and Human Services; 2019. https://aspe.hhs.gov/system/files/pdf/263361/improving-data-capacity-aian.pdf

[39] Indian Health Service. Agency overview. Rockville, MD: US Department of Health and Human Services, Indian Health Service. https://www.ihs.gov/aboutihs/overview

[40] Kramer BJ, Vivrette RL, Satter DE, Jouldjian S, McDonald LR. Dual use of veterans health administration and Indian Health Service: healthcare provider and patient perspectives. J Gen Intern Med 2009;24:758–64. 10.1007/s11606-009-0962-4 19381730 PMC2686768

[41] US Commission on Civil Rights. Broken promises: continuing federal funding shortfall for Native Americans. Washington, DC: US Commission on Civil Rights; 2018. https://www.usccr.gov/pubs/2018/12-20-Broken-Promises.pdf

[42] Call KT, McAlpine DD, Johnson PJ, Beebe TJ, McRae JA, Song Y. Barriers to care among American Indians in public health care programs. Med Care 2006;44:595–600. 10.1097/01.mlr.0000215901.37144.94 16708009

[43] Bachman R, Zaykaowski H, Kallmyer R, Poteyeva M, Lanier C. Violence against American Indian and Alaska Native women and the criminal justice response: what is known. Washington, DC: US Department of Justice; 2008. https://www.ojp.gov/pdffiles1/nij/grants/223691.pdf

[44] Greenfeld LA, Smith SK. American Indians and crime. Washington, DC: US Department of Justice, Bureau of Justice Statistics; 1999. https://www.bjs.gov/content/pub/pdf/aic.pdf

[45] Spencer CM, Stith SM. Risk factors for male perpetration and female victimization of intimate partner homicide: a meta-analysis. Trauma Violence Abuse 2020;21:527–40. 10.1177/1524838018781101 29888652

[46] Smith SG, Fowler KA, Niolon PH. Intimate partner homicide and corollary victims in 16 states: National Violent Death Reporting System, 2003–2009. Am J Public Health 2014;104:461–6. 10.2105/AJPH.2013.301582 24432943 PMC3953789

[47] Gray B, Lauderdale P. The web of justice: restorative justice has presented only part of the story. Wicazo Sa Rev 2006;21:29–41. 10.1353/wic.2006.0006

[48] Peacock T, George L, Wilson A, Bergstrom A, Pence E. Community-based analysis of the US legal system’s intervention in domestic abuse cases involving indigenous women. Washington, DC: US Department of Justice; 2003. https://www.ojp.gov/pdffiles1/nij/grants/199358.pdf

[49] H.R. 6545—Violence Against Women Reauthorization Act of 2018. 115th Congress. Washington, DC; 2018. https://www.congress.gov/bill/115th-congress/house-bill/6545/

[50] Violence Against Women Reauthorization Act of 2013. 113th Congress. Washington, DC; 2013. https://www.congress.gov/113/plaws/publ4/PLAW-113publ4.pdf

[51] Yucupicio P, Urbina F, Flores O. Pascua Yaqui Tribe VAWA Implementation. https://www.ncai.org/tribal-vawa/pilot-project-itwg/Pascua_Yaqui_VAWA_Pilot_Project_Summary_2015.pdf

[52] Henson M, Sabo S, Trujillo A, Teufel-Shone N. Identifying protective factors to promote health in American Indian and Alaska Native adolescents: a literature review. J Prim Prev 2017;38:5–26. 10.1007/s10935-016-0455-2 27826690 PMC5313316

[53] Cross T. Relational worldview model. Pathways Practice Digest 1997;12;4:6–7. https://www.sprc.org/sites/default/files/resource-program/Relational-Worldview-Model.pdf

[54] CDC. Technical packages for violence prevention: using evidence-based strategies in youth violence prevention efforts. Atlanta, GA: US Department of Health and Human Services, CDC; 2016–2017. https://www.cdc.gov/violenceprevention/communicationresources/pub/technical-packages.html

[55] Niolon PH, Kearns M, Dills J, Preventing intimate partner violence across the lifespan: a technical package of programs, policies and practices. Atlanta, GA: US Department of Health and Human Services, CDC; 2017. https://www.cdc.gov/violenceprevention/pdf/ipv-technicalpackages.pdf

[56] Foshee VA, Reyes LM, Agnew-Brune CB, The effects of the evidence-based Safe Dates dating abuse prevention program on other youth violence outcomes. Prev Sci 2014;15:907–16. 10.1007/s11121-014-0472-4 24599482 PMC11058563

[57] Crooks CV, Exner-Cortens D, Siebold W, The role of relationships in collaborative partnership success: lessons from the Alaska Fourth R project. Eval Program Plann 2018;67:97–104. 10.1016/j.evalprogplan.2017.12.007 29289925

[58] Schanen JG, Skenandore A, Scow B, Hagen J. Assessing the impact of a healthy relationships curriculum on Native American adolescents. Soc Work 2017;62:251–8. 10.1093/sw/swx021 28460025

[59] Hagen JW, Skenandore AH, Scow BM, Schanen JG, Clary FH. Adolescent pregnancy prevention in a rural Native American community. J Fam Soc Work 2012;15:19–33. 10.1080/10522158.2012.640926

[60] Armstead TL, Rambo K, Kearns M, Jones KM, Dills J, Brown P. CDC’s DELTA FOCUS program: identifying promising primary prevention strategies for intimate partner violence. J Womens Health (Larchmt) 2017;26:9–12. 10.1089/jwh.2016.6251 28099073 PMC5808986

[61] Multnomah County. Youth violence prevention. Portland, OR: Multnomah County; 2020. https://www.multco.us/health/community-health/youth-violence-prevention

[62] Coker AL, Fisher BS, Bush HM, Evaluation of the Green Dot bystander intervention to reduce interpersonal violence among college students across three campuses. Violence Against Women 2015;21:1507–27. 10.1177/1077801214545284 25125493 PMC5875923

[63] Messing JT, Campbell J, Wilson JS, Brown S, Patchell B, Shall C. Police departments’ use of the Lethality Assessment Program: a quasi-experimental evaluation. Washington, DC: US Department of Justice; 2014. https://www.ojp.gov/pdffiles1/nij/grants/247456.pdf

[64] Casteel C, Peek-Asa C. Effectiveness of crime prevention through environmental design (CPTED) in reducing robberies. Am J Prev Med 2000;18(Suppl):99–115. 10.1016/S0749-3797(00)00146-X 10793286

[65] Cook P, MacDonald J. Public safety through private action: an economic assessment of BIDs. Econ J (Lond) 2011;121:445–62. 10.1111/j.1468-0297.2011.02419.x

[66] MacDonald J, Golinelli D, Stokes RJ, Bluthenthal R. The effect of business improvement districts on the incidence of violent crimes. Inj Prev 2010;16:327–32. 10.1136/ip.2009.024943 20587814 PMC2976613

[67] Levitie J, Koulish J. State earned income tax credits: 2008 legislative update. Washington, DC: Center on Budget and Policy Priorities; 2008. https://www.cbpp.org/research/state-earned-income-tax-credits-2008-legislative-update?fa=view&id=462

[68] Center on Budget and Policy Priorities. Policy basics: the earned income tax credit. Washington, DC: Center on Budget and Policy Priorities; 2016. https://www.cbpp.org/research/federal-tax/policy-basics-the-earned-income-tax-credit

[69] Akee RK, Copeland WE, Keeler G, Angold A, Costello EJ. Parents’ incomes and children’s outcomes: a quasi-experiment. Am Econ J Appl Econ 2010;2:86–115. 10.1257/app.2.1.86 20582231 PMC2891175

[70] Dankovchik J, Hoopes MJ, Warren-Mears V, Knaster E. Disparities in life expectancy of Pacific Northwest American Indians and Alaska natives: analysis of linkage-corrected life tables. Public Health Rep 2015;130:71–80. 10.1177/003335491513000109 25552757 PMC4245288

[71] Hagen LA. Violent crime in Indian country and the federal response. Department of Justice Journal of Federal Law and Practice 2021;69:79–129.

[72] Pierce AS. American Indian adolescent girls: vulnerability to sex trafficking, intervention strategies. Am Indian Alsk Native Ment Health Res 2012;19:37–56. 10.5820/aian.1901.2012.37 22569724

[73] Farley M, Deer S, Golding JM, The prostitution and trafficking of American Indian/Alaska Native women in Minnesota. Am Indian Alsk Native Ment Health Res 2016;23:65–104. 10.5820/aian.2301.2016.65 28562843

[74] Deer S. Relocation revisited: sex trafficking of native women in the United States. William Mitchell Law Rev 2010;36.

